# Diabetes and atrial fibrillation in hospitalized patients in the United States

**DOI:** 10.1002/clc.23533

**Published:** 2021-02-04

**Authors:** Nilay Kumar, Justin B. Echouffo‐Tcheugui

**Affiliations:** ^1^ Division of Hospital Medicine, Department of Medicine University of Wisconsin School of Medicine Madison Wisconsin USA; ^2^ Department of Medicine Johns Hopkins University Baltimore Maryland USA

**Keywords:** atrial fibrillation, diabetes mellitus, trends

## Abstract

**Background:**

Data on the burden of atrial fibrillation (AF) associated with diabetes among hospitalized patients are scarce. We assessed the AF‐related hospitalizations trends in patients with diabetes, and compared AF outcomes in patients with diabetes to those without diabetes.

**Hypothesis:**

AF‐related health outcomes differ between patient with diabetes and without diabetes.

**Methods:**

Using the National Inpatient Sample (NIS) 2004–2014, we studied trends in AF hospitalization rate among diabetic patients, and compared in‐hospital case fatality rate, length of stay (LOS), cost and utilization of rhythm control therapies, and 30‐day readmission rate between patients with and without diabetes. Logistic or Cox regression models were used to assess the differences in AF outcomes by diabetes status.

**Results:**

Over the study period, there were 4 325 522 AF‐related hospitalizations, of which 1 075 770 (24.9%) had a diagnosis of diabetes. There was a temporal increase in AF hospitalization rate among diabetic patients (10.4 to 14.4 per 1000 hospitalizations among patients with diabetes; +4.4% yearly change, *p*‐trend < .0001). Among AF patients, those with diabetes had a lower in‐hospital mortality (adjusted odds ratio [aOR]: 0.68; 95% CI: 0.65–0.72) and LOS (aOR: 0.95; 95% CI: 0.94–0.96), but no difference in costs (aOR: 0.95; 95% CI: 0.94–0.96) and a higher 30‐day rate of readmissions compared with no diabetes (aHR 1.05; 95% CI: 1.01–1.08), compared to individuals without diabetes.

**Conclusion:**

AF and diabetes coexist among hospitalized patients, with rising trends over the last decade. Diabetes is associated with lower rates in‐hospital adverse AF outcomes, but a higher 30‐day readmission risk.

## 1 INTRODUCTION

Atrial fibrillation (AF) is the most common arrhythmia in the United States,^1^, ^2^, ^3^ with AF cases projected to increase to 15.9 million by the year 2050.^1^, ^2^ The US burden of diabetes has also been growing,^4^ with a projected 30% or more increase by 2050.^5^ Diabetes increases the risk of AF by ~40%.^6^ Diabetes and AF often coexist, posing unique clinical challenge.^7^ There is a paucity of US national data on the patterns and time trends of coexisting AF and diabetes. Insights into the burden of coexisting diabetes and AF may help in improving outcomes, as diabetes may adversely influence AF outcomes.^8^ The link between diabetes and AF also has potential implications for diabetes screening among those with AF patients and vice versa, and a potential therapeutic implication. Emerging trial data suggests that sodium glucose cotransporter (SGLT)‐2 inhibitors may have beneficial effects on AF outcomes among individuals with diabetes.^9^

Using the Nationwide Inpatient Sample (NIS) registry data, we investigated: (1) the trends in AF hospitalization rate, in‐hospital case fatality rate, average length of stay (LOS), cost and utilization of rhythm control therapies among patients with diabetes; and (2) the potential differences in in‐hospital mortality, LOS, cost, utilization of rhythm control therapies, and 30‐day readmission rates between hospitalized AF patients with and without diabetes.

## 2 METHODS

### 2.1 Data sources and study population

Our study was based on data from the National Inpatient Sample (NIS) covering the 2004–2014 period. The NIS is the largest all‐payer database of inpatient hospital stays in the United States with approximately 35 million weighted hospitalization records per year. The NIS is developed by the Agency for Healthcare Research and Quality (AHRQ) as part of its Healthcare Cost and Utilization Project (HCUP). Briefly, the NIS is a 20% stratified sample of inpatient hospitalization from participating states. It is representative of >95% of all inpatient hospitalizations in the United States.^10^ Prior to 2012, the NIS comprised all hospitalizations from a 20% stratified sample of community hospitals (defined as all non‐federal hospitals with the exception of rehabilitation facilities). In 2012, the NIS underwent a redesign and is now comprised of a 20% stratified systemic sample of discharges from all community hospitals from participating states.^11^ This design has been shown to decrease SE estimates while calculating variances at the national level and make these estimates more generalizable to the target universe. To facilitate multi‐year trend analysis and ensure comparability of estimates, the AHRQ has published *trend weights* using the 2012 methodology on previous years.^12^ The details of the NIS sampling methodology are published annually by the AHRQ. Due to the de‐identified nature of this data, the study was exempt from Institutional Review Board approval from the primary institution.

To assess readmissions among patients with a primary diagnosis of AF discharged between January and November 2014, we used the 2014 Nationwide Readmissions Database (NRD), developed by the AHRQ as part of the HCUP. The NRD is the largest all‐payer database of hospital readmissions in the United States. In order to support readmission analyses, the NRD contains a linkage variable (*NRD_visitlink*) by means of which patients can be tracked across hospitalizations. The target universe for the NRD includes inpatient discharges from community hospitals that were not rehabilitation or long‐term acute care facilities. The sampling frame for the NRD includes inpatient discharges from participating states, excluding rehabilitation and LTAC facilities. All discharges from the sampling frames are included. Discharge weights for the NRD were developed with the target universe as the standard. We included hospitalized patients aged ≥18 years in both the trends and readmissions analyses.

### 2.2 Atrial fibrillation and diabetes ascertainment

A primary diagnosis of AF was based on the *International Classification of Diseases, Ninth Revision* (ICD‐9) code 427.31. Diabetes was ascertained using the *Clinical Classification Software* (CCS) codes 49 and 50. Prior studies have shown that the use of ICD‐9‐CM coding has a high degree of accuracy for identifying atrial fibrillation^13^, ^14^ and diabetes mellitus,^15^ with a robust sensitivity and an excellent sensitivity and specificity (both >90% for each condition). The complete list of codes using in the study are summarized in the Table S1.

Since the NIS and Nationwide Readmissions Database (NRD) are both administrative databases, we are able to identify a diagnosis of diabetes only by the use of ICD‐9 CM diagnosis and procedure codes. There are over 50 ICD‐9 CM codes for diabetes with or without complications (250.xx). The CCS coding system is developed by the AHRQ and maps similar ICD‐9 CM codes to a manageable number of CCS codes (https://www.hcup-us.ahrq.gov/toolssoftware/ccs/ccsfactsheet.jsp). We chose to use CCS codes for defining diabetes mellitus because using 2 CCS codes rather than >50 ICD‐9 CM codes makes our analysis less prone to errors. The CCS coding system is designed to capture all of the disparate ICD‐9 codes that are used to identify diabetes in administrative data.

### 2.3 Outcomes

The study outcomes included hospitalization rate per 1000 hospitalizations related to diabetes, in‐hospital outcomes (in‐hospital case fatality rate, LOS, cost, utilization or rhythm control therapies), and 30‐day all‐cause readmission.

The rhythm control therapies included radiofrequency catheter ablation and electrical cardioversion, identified using relevant *ICD‐9* and *CCS* procedure codes (Table S1). Since codes for ablation and cardioversion are not specific to AF and could be used for other arrhythmias, we excluded records with a diagnosis of other atrial or ventricular arrhythmias as having had AF ablation or electrical cardioversion. We also excluded records with implantation of permanent pacemaker during the same hospitalization because the ablation codes may represent a rate‐controlled strategy with atrioventricular (AV) nodal ablation and pacemaker implantation.

### 2.4 Statistical analysis

We assessed the yearly prevalence of AF and estimates of the length of hospital stay and costs, and the related time trends, among patients with diabetes based on the year of admission in all patients, across four age groups, (18–45 years, 45–64 years, 65–74 years and ≥75 years) and by sex. Survey analysis techniques were used to produce national estimates as recommended by the AHRQ. Continuous variables were expressed as mean (SE) while categorical variables were expressed as percentages. Differences in continuous and categorical variables between the diabetes and no‐diabetes groups were tested using linear and logistic regression models. Linear trends in continuous as well as categorical variables were examined using linear regression with year as a continuous predictor. We examined overall and subgroup specific trends in AF hospitalization rate per thousand diabetes‐related hospitalizations, in‐hospital case fatality rate, LOS, cost and utilization of rhythm control therapies. The costs were estimated by multiplying total charges with hospital cost‐to‐charge ratios supplied by the AHRQ. Costs were adjusted to 2014 US dollars using consumer price index inflation adjustment calculator from the Bureau of Labor Statistics.^16^

The differences in in‐hospital AF outcomes between the diabetes and no‐diabetes groups of patients were assessed using logistic regression models. The latter included the following adjustment variables: age, sex, income quartile, Charlson comorbidity index, hypertension, chronic renal failure, congestive heart failure, obesity, peripheral vascular disease, deficiency anemia, chronic lung disease, pulmonary circulation disorders, coagulopathy, rhythm control procedure (catheter ablation and electrical cardioversion), hospital region, hospital location and teaching status.

The differences in all‐cause 30‐day readmission among AF patients with and without diabetes were examined using a Kaplan–Meier curve and the log‐rank test. A multivariable adjusted Cox proportional hazards model was also used with adjustment for age, sex, income quartile, Charlson comorbidity index, hypertension, chronic renal failure, congestive heart failure, obesity, peripheral vascular disease, deficiency anemia, chronic lung disease, pulmonary circulation disorders, coagulopathy, rhythm control procedure (catheter ablation and electrical cardioversion), hospital region, hospital location and teaching status.

A *p*‐value of ≤.05 was considered significant. All analyses were conducted using Stata 15.1 statistical package (Statacorp, College Station, TX).

## 3 RESULTS

### 3.1 Trends in atrial fibrillation hospitalization in patients with diabetes mellitus

The weighted total of hospitalizations with a primary diagnosis of AF during the study period was 4 325 522, of which 1 075 770 (24.9%) had a diagnosis of diabetes. Mean age of patients with primary diagnosis of AF in the setting of diabetes was 70.4 years (SE 0.04) and 51.8% were women. There was a declining trend in the proportion of females (53%–50.5%, *p*‐trend < .001). There was an overall increase in comorbidity burden (mean Charlson comorbidity index 2.0 [SE 0.01] to 2.5 [SE 0.01], *p*‐trend < .001). Other trends in baseline characteristics are detailed in Table 1.

**TABLE 1:** Trends in baseline characteristics for atrial fibrillation hospitalizations in the setting of coexisting diabetes mellitus

Baseline characteristics	2004–2006 (weighted *n* = 208 042)	2007–2008 (weighted *n* = 180 042)	2009–2010 (weighted *n* = 211 419)	2011–2012 (weighted *n* = 239 256)	2013–2014 (weighted *n* = 236 195)	Overall (weighted *n* = 1 075 770)	*p*‐Trend
Age, mean (SE)	70.5 (0.08)	70.3 (0.09)	70.5 (0.10)	70.5 (0.08)	70.3 (0.06)	70.3 (0.04)	.99
Female (%)	53.0	52.1	51.9	51.6	50.5	51.8	<.001
Age group (%)							
18–44 years	2.2	2.4	2.1	2.1	2.0	2.1	.01
45–64 years	27.7	27.9	28.0	27.7	28.0	27.9	.59
65–74 years	28.9	29.3	29.7	30.2	31.1	29.9	<.001
≥75 years	41.3	40.4	40.2	40.0	38.9	40.1	<.001
Race (%)							
White	57.6	59.8	67.4	72.5	74.6	66.8	<.001
Black	6.8	7.1	8.1	9.2	9.4	8.2	<.001
Hispanic	5.4	5.4	6.1	6.6	6.9	6.2	<.001
Asian or Pacific Islander	1.2	1.5	1.5	1.6	1.7	1.5	.001
Native American	0.4	0.6	0.6	0.6	0.5	0.5	.25
Others	1.5	1.9	2.1	2.1	2.1	2.0	.002
SES (%)							
Quartile 1	28.1	28.1	28.0	30.1	29.9	28.9	.003
Quartile 2	27.3	28.4	28.2	26.3	28.7	27.8	<.001
Quartile 3	23.8	24.0	24.6	24.5	23.3	24.0	.63
Quartile 4	20.8	19.5	19.2	19.1	18.1	19.3	.002
Primary payer (%)							
Medicare	70.1	68.5	68.5	70.9	70.8	69.9	<.001
Medicaid	4.5	4.3	5.0	4.8	5.7	4.9	<.001
Private insurance	21.1	22.6	21.6	19.5	18.7	20.7	<.001
Self pay/uninsured	2.3	2.3	2.7	2.6	2.5	2.5	.20
AHRQ comorbidity measure							
AIDS (%)	0.03	0.04	0.04	0.04	0.04	0.04	.91
Alcohol abuse (%)	2.0	2.4	2.6	3.1	3.3	2.7	<.001
Deficiency anemias (%)	10.9	13.7	15.4	17.3	17.5	15.1	<.001
Rheumatoid arthritis/Collagen vascular diseases (%)	1.9	2.2	2.4	2.6	2.9	2.4	<.001
Chronic blood loss anemia (%)	0.6	0.6	0.5	0.6	0.5	0.6	.10
Congestive heart failure (%)	0.5	0.4	0.6	0.4	0.4	0.5	.15
COPD (%)	21.8	22.8	24.2	25.8	26.8	24.4	<.001
Coagulopathy (%)	2.2	2.6	3.3	3.7	4.0	3.2	<.001
Depression (%)	6.0	7.5	8.5	9.6	10.2	8.5	<.001
Diabetes, uncomplicated (%)	87.5	83.5	83.1	81.7	80.8	83.2	<.001
Diabetes with chronic complications (%)	10.7	10.8	10.6	11.5	12.0	11.2	<.001
Drug abuse (%)	0.5	0.6	0.7	1.1	1.4	0.9	<.001
Hypertension (%)	69.8	73.4	76.7	79.4	80.7	76.3	<.001
Hypothyroidism (%)	12.7	14.6	15.7	16.9	17.3	15.6	<.001
Liver disease (%)	1.2	1.4	1.8	2.0	2.5	1.8	<.001
Fluid and electrolyte disorders (%)	15.7	17.8	20.0	22.2	24.7	20.3	<.001
Other neurological disorders (%)	4.0	5.1	5.7	6.1	6.2	5.4	<.001
Obesity (%)	13.5	18.5	21.3	25.0	28.1	21.6	<.001
Paralysis (%)	1.3	1.5	1.4	1.5	1.4	1.4	.12
Peripheral vascular disease (%)	7.0	7.7	8.4	9.2	9.4	8.4	<.001
Psychoses (%)	1.7	2.1	2.3	2.9	2.9	2.4	<.001
Pulmonary circulation disorders (%)	0.04	0.1	0.2	0.1	0.1	0.1	<.001
Renal failure (%)	10.4	15.4	18.2	21.2	22.7	17.9	<.001
Solid tumor without metastasis (%)	1.9	1.9	2.0	2.1	2.1	2.0	.07
Valvular disease (%)	0.2	0.2	0.2	0.2	0.2	0.2	.16
Weight loss (%)	1.0	1.3	1.7	2.3	2.3	1.8	<.001
CCI, mean (SE)	2.0 (0.01)	2.2 (0.01)	2.3 (0.01)	2.4 (0.01)	2.5 (0.01)	2.29 (0.004)	<.001
Hospital location and teaching status							
Rural	16.6	15.7	15.4	14.6	13.2	15.0	<.001
Urban non‐teaching	46.0	45.2	45.6	42.3	34.7	42.5	<.001
Urban teaching	37.5	39.1	39.0	43.1	52.0	42.5	<.001
Hospital Region							
Northeast	20.8	19.1	20.5	19.6	19.0	19.8	.06
Midwest	25.4	26.1	25.7	24.5	24.9	25.3	.14
South	39.7	40.1	39.2	41.0	41.4	40.3	.04
West	14.1	14.8	14.6	14.9	14.7	14.6	.26

Abbreviations: AHRQ, Agency for Healthcare Research and Quality; CCI, chronic condition, indicator.

The rate of AF hospitalizations increased from 10.4 to 14.4 per thousand diabetes‐related hospitalizations from 2004 to 2014 (*p*‐trend < .001). This upward trend was evident across all age groups and sexes (Table 2). In‐hospital case fatality rate improved modestly from 1.1% in 2004 to 0.9% 2014 (*p*‐trend = .03). In subgroup analyses, this trend was not evident in the various age groups, except for the ≥75 years age group. In subgroup analyses by sex, there was no clear downward trend in females and males (Table 2). In multivariable analyses, the improvement in case fatality rate was not fully explained by changing trends in age, sex and comorbidities (Table S2).

**TABLE 2:** Trends in characteristics of hospitalization for atrial fibrillation among patients with diabetes mellitus

Year	2004	2005	2006	2007	2008	2009	2010	2011	2012	2013	2014	Linear slope (% change/year)	*p*‐Trend
Hospitalization rates (per 1000)													
Overall	10.4	10.9	11.6	12.1	13.1	13.7	13.6	14.4	15.0	14.9	14.4	+ 4.4	<.001
18–44 years	2.1	2.3	2.5	2.9	2.9	2.8	2.8	3.2	3.1	2.9	2.9	+3.9	<.001
45–64 years	8.0	8.8	9.5	9.9	10.3	10.8	10.5	11.3	11.6	11.9	11.4	+4.3	<.001
65–74 years	12.8	13.1	13.7	15.0	16.2	16.8	17.3	17.7	18.5	18.5	17.6	+4.6	<.001
≥75 years	14.2	14.3	15.2	15.7	17.2	18.3	18.2	19.0	20.0	19.3	19.5	+4.4	<.001
Males	10.4	11.1	11.7	12.4	13.3	13.8	13.8	14.5	15.2	15.1	14.6	+4.4	<.001
Females	10.4	10.7	11.4	11.9	12.9	13.7	13.4	14.3	14.8	14.7	14.2	+4.3	<.001
Fatality rates													
Overall	1.1	1.1	1.1	0.9	0.9	0.9	0.9	0.9	0.9	1.0	0.9	−1.4	.03
18–44 years	0	0	0	0.5	0.2	0.5	0.2	0.2	0.2	0	0.2	0	.85
45–64 years	0.7	0.5	0.3	0.4	0.5	0.3	0.4	0.3	0.4	0.5	0.6	0	.94
65–74 years	1.0	0.7	0.7	0.8	0.7	0.6	0.6	0.6	0.8	0.8	0.8	−0.3	.76
≥75 years	1.5	1.8	1.9	1.4	1.4	1.4	1.5	1.5	1.3	1.6	1.2	−2.1	.02
Males	1.0	0.9	0.9	0.9	0.8	0.8	0.7	0.7	0.9	0.9	0.8	−1.0	.30
Females	1.2	1.2	1.2	0.8	1.0	0.9	1.0	1.0	0.9	1.1	1.0	−1.6	.06
Length of stay													
Overall	4.0	4.0	4.0	3.9	3.9	3.9	3.8	3.7	3.8	3.7	3.7	−0.8	<.001
18–44 years	2.9	3.1	2.8	3.1	3.1	2.7	3.0	2.8	2.9	3.0	2.9	−0.3	.59
45–64 years	3.5	3.6	3.6	3.5	3.5	3.5	3.4	3.4	3.4	3.4	3.4	−0.7	<.001
65–74 years	3.9	3.8	3.9	3.8	3.8	3.9	3.7	3.7	3.6	3.6	3.7	−0.8	<.001
≥75 years	4.4	4.4	4.4	4.3	4.2	4.3	4.3	4.1	4.1	4.1	4.1	−0.8	<.001
Males	3.7	3.8	3.8	3.7	3.6	3.7	3.6	3.6	3.6	3.6	3.6	−0.8	<.001
Females	4.2	4.2	4.2	4.1	4.1	4.1	4.0	3.9	3.9	3.9	3.9	−0.9	<.001
Costs (USD)													
Overall	9810	10 228	10 615	10 548	10 410	10 605	10 489	10 591	9356	9252	9125	−1.3	<.001
18–44 years	7990	8579	8823	10 339	9638	8614	9420	8755	8347	8068	7976	−1.3	.05
45–64 years	9438	10 076	10 524	10 532	10 557	10 665	10 337	10 814	9515	9172	9221	−1.1	<.001
65–74 years	9887	10 210	10 641	10 757	10 566	10 783	10 665	10 914	9537	9549	9515	−1.0	<.001
≥75 years	10 076	10 426	10 760	10 420	10 239	10 535	10 520	10 293	9161	9130	8808	−1.7	<.001
Males	9779	10 538	10 895	11 075	10 628	10 985	10 800	11 058	9813	9522	9458	−1.2	<.001
Females	9838	9948	10 367	10 066	10 211	10 258	10 199	10 159	8922	8989	8797	−1.4	<.001

We observed improvements in LOS (mean of 4.0 days in 2004 to 3.7 days in 2014, *p*‐trend < .001). This trend was significant across all but the youngest age subgroup (Table 2). Similarly, the average cost declined from $9810 in 2004 to $9125 in 2014 (*p*‐trend < .001); and this trend was significant across all subgroups (Table 2).

The utilization of radiofrequency ablation and electrical cardioversion increased linearly in both the diabetes and no‐diabetes groups of patients (*p*‐trend < .001, Figures S1 and S2).

### 3.2 Outcomes of atrial fibrillation in patients with and without diabetes

The differences in baseline characteristics by diabetes status for patients admitted with a primary diagnosis of AF are summarized in Table S3. Patients with diabetes were older (70.4 vs. 70.2 years, *p* < .001), with a lower proportion of females (51.8% vs. 52.4%, *p* < .001), and a higher burden of comorbidities (Charlson comorbidity index 2.29 vs. 0.99, *p* < .001), compared to patients without diabetes.

In multivariable adjusted models (Table 3), diabetes was associated with lower in‐hospital mortality (adjusted odds ratio [aOR]: 0.68; 95% CI: 0.65–0.72); lower LOS (aOR: 0.95; 95% CI: 0.94–0.96), lower rates of utilization of radiofrequency ablation (aOR: 0.96; 95% CI: 0.92–0.99) and electrical cardioversion (aOR: 0.92; 95% CI: 0.89–0.94). There was no significant difference in costs (aOR: 0.95; 95% CI: 0.94–0.96).

**TABLE 3:** Outcomes of atrial fibrillation hospitalization among patients with diabetes compared to those without diabetes

Outcomes	Unadjusted OR (95% CI)	*p*‐Value	Adjusted OR (95% CI)^a^	*p*‐Value
In‐hospital outcomes				
In‐hospital mortality	0.95 (0.90–0.99)	.04	0.68 (0.65–0.72)	<.001
LOS > median	1.37 (1.36–1.39)	<.001	0.95 (0.94–0.96)	<.001
Cost > median	1.27 (1.25–1.28)	<.001	0.99 (0.98–1.00)	.11
Catheter ablation	0.65 (0.62–0.68)	<.001	0.96 (0.92–0.99)	.04
Direct current cardioversion	0.86 (0.84–0.88)	<.001	0.92 (0.89–0.94)	<.001
Post‐discharge outcomes	Unadjusted HR (95% CI)		Adjusted HR (95% CI)^a^	
30‐day readmission rate	1.33 (1.29–1.37)	<.001	1.05 (1.01–1.08)	.01

Abbreviations: CI, confidence interval; HR, hazard ratio; LOS, length of stay; OR, odds ratio.

^a^ Estimates are adjusted for age, sex, and socioeconomic status, Charlson comorbidity index, comorbid conditions (congestive heart failure, chronic lung disease, hypertension, obesity, peripheral vascular disease, valvular disease, chronic kidney disease, Pulmonary circulation disorders, coagulopathy), and rhythm control procedure.

There were a total 359 199 index admissions for a primary diagnosis of AF in 2014 (mean age: 70.4 years [SE: 0.09], 51.8% women), among whom 28.9% had diabetes, 71.8% had a diagnosis of hypertension and 0.3% had a diagnosis of congestive heart failure. The overall rate of all‐cause 30‐day readmission was 15.2%. A higher proportion of patients with diabetes had at least one readmission event within 30 days (18.1% vs. 14.0%, *p* < .001).

In time‐to‐event analysis (Figure S3 and Table 3), diabetes was associated with significantly higher rates of all‐cause 30‐day readmission compared with no diabetes (adjusted HR 1.05, 95% CI: 1.01–1.08, *p* = .01).

### 3.3 30‐day readmissions after an atrial fibrillation hospitalization in patients with and without diabetes

We observed significant differences in causes for readmission between those with diabetes compared to those without diabetes. Of the patients who were readmitted, a greater proportion of patients with diabetes had a primary diagnosis of congestive heart failure or sepsis compared with patients without diabetes (All *p <* .01), while a lower proportion of patients with diabetes had a primary diagnosis of cardiac dysrhythmias compared with patients without diabetes (Figure S4).

## 4 DISCUSSION

This study reports data on diabetes and AF comorbidities over a 10‐year period in the United States, using a large nationwide sample from the NIS registry. We made several observations. A principal diagnosis of atrial fibrillation was more frequent among patients hospitalized for diabetes over time. Conversely, of all hospitalizations for a principal diagnosis of AF, nearly a quarter had coexistent diabetes. Over the 2004–2014 period, there was an increase in the frequency of AF‐related hospitalizations among patients with diabetes across all age groups, but with a decrease in in‐hospital fatality rate, length of hospital stay and related‐costs. Among AF patients, compared to those without diabetes, patients with diabetes had a shorter hospital stay with lower costs, and a lower in‐hospital mortality, but a higher 30‐day readmission rate.

Despite accruing evidence on diabetes as an important precursor of AF,^6^ studies have seldom examined the trends of comorbid AF and diabetes. A prior analysis of the 2000–2010 NIS data showed that the proportion of individuals with diabetes among AF patients has been increasing over time,^17^ but this study did not focused on diabetes. We described an increasing prevalence of concomitant AF and diabetes over the 2004–2014 period. The latter trend may be related to an increase in the frequency of AF (and its detection) over time possibly due to an aging population. This could also be related to an intrinsic increase in diabetes prevalence,^18^ as well as a surge in the number of individuals diagnosed with diabetes as a result of the adoption of HbA_1C_ as an additional diagnostic test since 2009.^19^ Our findings of decreasing trends in AF‐related LOS and case‐fatality in the hospital maybe related to an improvement of AF care in general, and among those with diabetes in particular. The latter is possibly related to the fact that diabetes is a component of the stroke risk calculators (CHADS2 and CHA2DS2‐VASc). Indeed, an important fraction of patients with AF and diabetes will have a stroke risk score that makes them eligible for anticoagulation therapy.^20^

A limited number of studies have evaluated the association between diabetes and clinical outcomes among individuals with AF.^8^, ^21^, ^22^, ^23^ Our study showed that diabetes is inversely related to in‐hospital mortality, length of hospital stay and costs among patients with AF, but is independently associated with readmissions. Prior studies have suggested that diabetes among patients with AF is associated with an increased risk of both mortality and readmission,^24^, ^25^ Indeed, in the ORBIT‐AF registry, diabetes was found to be significantly associated with all‐cause hospitalizations.^24^ It is important to indicate that in contrast to our investigation that examined in‐hospital outcomes (including mortality) between patients with and without diabetes, the prior studies mainly examined longer term mortality.^8^, ^21^, ^22^, ^23^ The inverse associations of diabetes with in‐hospital mortality, length of stay and costs, as may be partially explained by an overall higher rate of use of thromboembolic events prevention among the admitted AF patients with diabetes as a diagnosis (good quality of acute [hospital‐based] AF care) and thus potentially better short‐term outcomes among patients with diabetes. This pattern most likely stems from the inclusion of diabetes in the stroke risk calculators (CHADS2 and CHA2DS2‐VASc). Indeed, prior studies have shown an association between diabetes and an increased use of targeted pharmacological therapies (anticoagulation, rhythm and rate control medications) among patients with AF,^8^, ^26^ which may be consistent with the lower use of AF‐related procedure as noticed in our investigation. In terms of the costs, the findings of lower costs associated with diabetes is also consistent with prior findings indicating that diabetes is not an important driver of AF related costs.^27^

Our findings have several important implications for clinical practice, as they highlight potential gaps respect to conjoint management of diabetes and AF, which may present unique challenges, especially in the context of constantly evolving diabetes and AF therapies. While new diabetes therapies (e.g., sodium‐glucose cotransporter‐2 inhibitors) have been emerging and showing important cardiovascular benefits, especially in terms of heart failure outcomes.^28^ While influence of these novel therapies on atrial fibrillation remains to be fully explored, early reports have showed that investigations suggest that dapagliflozin significantly decreases the incidence of atrial fibrillation/flutter episodes among diabetes patients.^9^

Hitherto, the association between diabetes mellitus and AF has remained under‐recognized by clinicians.^7^ Our findings strengthen the potential need for active screening for diabetes among people with AF, or vice‐versa; which may have a significant effect on reducing healthcare costs as suggested by our findings. Addressing diabetes in relation to AF may have a significant population‐level impact, as suggested by a population‐based study showing that ~5% of incident AF cases are attributable to diabetes.^6^

The strengths of our study include a large multiethnic sample with a nationwide scope, a standardized data collection methodology, and the exploration of a variety of AF‐related outcomes. However, there are limitations to our study. First, the NIS registry only captures in‐hospital data, and thus may have not captured individuals in the community with both diabetes and AF. Second, we did not examine the subtypes of AF and its pharmacological therapies (anticoagulation, rhythm and rate control drugs), which may both may have a different distribution among those with diabetes and could be associated with outcomes. Third, given the reliance on clinical practice to ascertain diabetes, we may have missed the patients without a prehospitalization diagnosis of diabetes and not tested during their hospital stay, especially as up to one‐fourth of all persons with diabetes in the United States are undiagnosed.^4^ The underestimation of diabetes may bias the estimate of the association of diabetes and outcomes (e.g., in‐hospital mortality) towards the null. Fourth, we did not assess the type and/or severity of diabetes, as well as therapies (insulin or noninsulin‐based therapies), which may influence glycemic control and AF outcomes. We also lacked information on glycosylated hemoglobin [HbA_1C_] that reflects the degree of blood glucose control. Indeed, extant evidence suggest that levels of HbA_1C_,^29^ and diabetes duration are determinants of the risk of stroke and thromboembolic events among patients with AF.^30^, ^31^ Fifth, given that we examined in‐hospital and short‐term outcomes, we may have not captured the full extent of the influence of diabetes on the outcomes of AF.

In conclusion, at least one in ten hospitalized patients with diabetes has atrial fibrillation, these proportion have been increasing with time. Approximately one quarter of AF patients have diabetes. In terms of atrial fibrillation outcomes, compared to those without diabetes, patients with diabetes tend to experience a shorter hospital stay and lower in‐hospital mortality, but a higher rate of readmission.

## Supporting information


**Data S1.** Supplementary Information.

## Data Availability

The data that support the findings of this study are available from the Agency of Health Care Research and Quality. Restrictions apply to the availability of these data, which were used under license for this study. Data are available at https://www.hcup-us.ahrq.gov/nisoverview.jsp with the permission of the Agency of Health Care Research and Quality.
