# Supplementary material for: Diabetes and atrial fibrillation in hospitalized patients in the United States
Source: Clin Cardiol. 2021 Feb 4;44(3):340–8. doi: 10.1002/clc.23533 (PMC7943913; doi:10.1002/clc.23533)
Supplement: Supplementary file 1 — Data S1. Supplementary Information. [file CLC-44-340-s001.docx]

**Diabetes and Atrial Fibrillation in Hospitalized Patients in the United States**

**Supplemental materials**

**Supplemental Table 1:** ICD-9 and Clinical classification software (CCS) codes used in the analysis

| **Variable** | **ICD-9 and/or CCS codes used** |
| --- | --- |
| Atrial fibrillation | ICD-9 code 427.31 |
| DM | CCS codes 49 and 50 |
| DC cardioversion | ICD-9 procedure code 99.61, CCS code 225 |
| AF ablation | ICD-9 procedure code 37.34 |
| Atrial flutter | ICD-9 code 427.32 |
| Non-paroxysmal AV nodal tachycardia | ICD-9 code 426.89 |
| Paroxysmal SVT | ICD-9 code 427.0 |
| Paroxysmal VT | ICD-9 code 427.1 |
| Ventricular premature beats | ICD-9 code 427.69 |
| Wolf-Parkinson-White syndrome | ICD-9 code 426.7 |
| Implantation of pacemaker in same hospitalization | ICD-9 procedure codes 37.80 – 37.83 |
| Cardiac dysrhythmias | CCS code 106 |
| CHF | CCS code 108 |
| Sepsis | CCS code 2 |
| Pneumonia | CCS code 122 |
| GI bleed | CCS code 153 |
| COPD | CCS code 127 |
| Acute kidney injury | CCS code 157 |
| Cerebrovascular accident | CCS code 109 |
| Respiratory failure | CCS code 131 |
| Device complication | CCS code 237 |

**Supplemental Table 2:** Yearly change in in-hospital case fatality after adjusting for baseline characteristics

| **Primary Atrial Fibrillation Hospitalizations in Patients with diabetes mellitus** | **Odds ratio of death per year**  OR (95% CI; p-value) |
| --- | --- |
| *Model 1*: unadjusted | 0.98 (0.97–0.99; 0.03) |
| *Model 2*: adjusted for age, sex | 0.98 (0.97–0.99; 0.03) |
| *Model 4*: age, sex, Charlson comorbidity index, hospital region, hospital location and teaching status, primary payer, SES | 0.95 (0.93–0.96; <0.001) |

**Supplemental Table 3:** Differences in baseline characteristics between atrial fibrillation hospitalizations with and without a diagnosis of diabetes mellitus

| **Baseline characteristics** | **Atrial fibrillation without diabetes**  Weighted n=3,249,752  proportion of all AF=75.1% | **Atrial fibrillation with diabetes**  weighted *n*=1,075,770  proportion of all AF=24.9 | ***P-value*** |
| --- | --- | --- | --- |
| Age, mean (SE) | 70.2 (0.04) | 70.4 (0.04) | <0.001 |
| Female (%) | 52.4 | 51.8 | <0.001 |
| **Race (%)** |  |  |  |
| White | 71.5 | 67.0 | <0.001 |
| Black | 5.3 | 8.2 | <0.001 |
| Hispanic | 3.9 | 6.2 | <0.001 |
| Asian or pacific islander | 1.1 | 1.5 | <0.001 |
| Other | 0.4 | 0.5 | <0.001 |
| **Primary payer (%)** | | | |
| Medicare | 65.1 | 69.9 | <0.001 |
| Medicaid | 3.7 | 4.9 | <0.001 |
| Private insurance | 25.9 | 20.6 | <0.001 |
| **SES (%)** | | | |
| Quartile 1 | 24.3 | 28.9 | <0.001 |
| Quartile 2 | 26.5 | 27.8 | <0.001 |
| Quartile 3 | 24.8 | 24.0 | <0.001 |
| Quartile 4 | 24.4 | 19.3 | <0.001 |
| **Comorbidities (%)** | | | |
| Hypertension | 61.1 | 76.2 | <0.001 |
| Chronic kidney disease | 8.7 | 17.9 | <0.001 |
| Congestive heart failure | 0.4 | 0.5 | <0.001 |
| Valvular disease | 0.2 | 0.2 | 0.23 |
| Coagulopathy | 2.8 | 3.2 | <0.001 |
| Peripheral vascular disease | 5.5 | 8.4 | <0.001 |
| Obesity | 9.1 | 21.6 | <0.001 |
| Chronic lung disease | 20.1 | 24.4 | <0.001 |
| Neurological diseases | 5.4 | 5.4 | 0.63 |
| Deficiency Anemia | 10.2 | 15.1 | <0.001 |
| Charlson comorbidity index mean (SE) | 0.99 (0.003) | 2.29 (0.004) | <0.001 |

**Supplemental Table 4:** Results of multivariate Cox proportional hazards model for all-cause 30-day readmission after hospitalization for a primary diagnosis of atrial fibrillation

| **Covariates** | **Hazard ratio (95% CI)** | **p-value** |
| --- | --- | --- |
| Diabetes mellitus | 1.05 (1.01 – 1.08) | 0.01 |
| **Demographics** |  |  |
| Age | 1.007 (1.005 – 1.008) | <0.001 |
| Female sex | 1.15 (1.12 – 1.19) | <0.001 |
| **Comorbidities** |  |  |
| Charlson comorbidity index | 1.18 (1.16 – 1.19) | <0.001 |
| CHF | 1.18 (0.95 – 1.46) | 0.14 |
| Chronic lung disease | 1.19 (1.15 – 1.24) | <0.001 |
| Hypertension | 1.00 (0.96 – 1.03) | 0.83 |
| Obesity | 0.99 (0.95 – 1.03) | 0.59 |
| Peripheral vascular disease | 1.04 (0.99 – 1.10) | 0.09 |
| Valvular disease | 0.93 (0.68 – 1.28) | 0.66 |
| Chronic kidney disease | 1.07 (1.03 – 1.12) | 0.002 |
| Pulmonary circulation disorders | 1.80 (1.32 – 2.46) | <0.001 |
| Coagulopathy | 1.15 (1.08 – 1.23) | <0.001 |
| **Rhythm control procedures** |  |  |
| AF ablation | 0.84 (0.75 – 0.94) | 0.002 |
| Electrical cardioversion | 0.90 (0.83 – 0.98) | 0.01 |
| **SES quartile** |  |  |
| First quartile | Reference |  |
| Second quartile | 0.91 (0.87 – 0.94) | <0.001 |
| Third quartile | 0.88 (0.84 – 0.92) | <0.001 |
| Fourth quartile | 0.85 (0.81 – 0.88) | <0.001 |


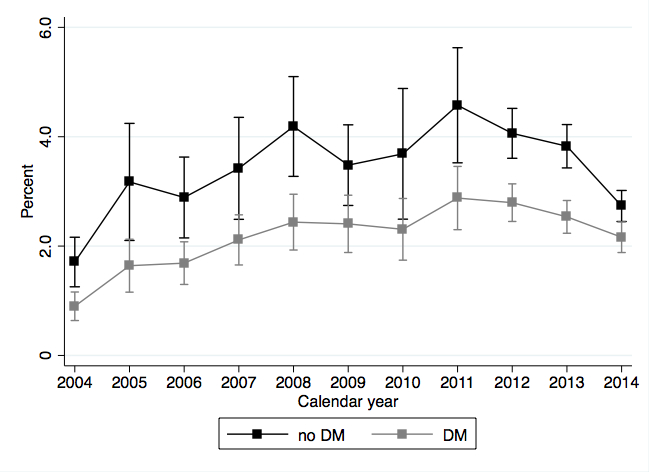


**Supplemental Figure 1:** Trends in utilization of catheter ablation among patients hospitalized for a primary diagnosis of AF stratified by diabetes (DM) diagnosis. There was a significant linear trend for patients without diabetes (P-trend<0.001, linear slope of +6.8% per year) as well as patients with diabetes (P-trend<0.001, linear slope +13% per year)


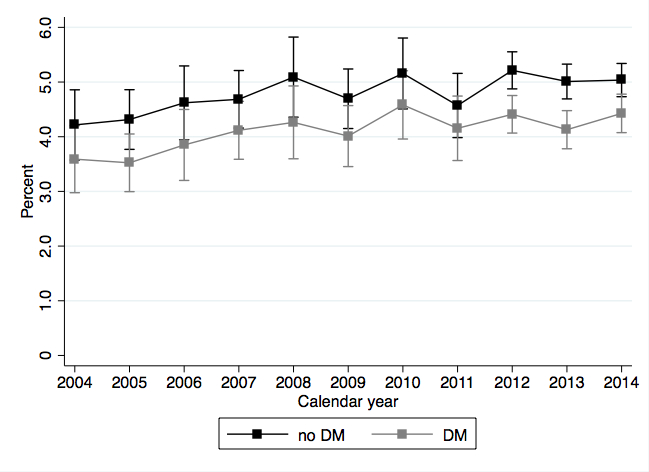


**Supplemental Figure 2:** Trends in electrical cardioversion among patients hospitalized with a primary diagnosis of AF stratified by diabetes (DM) diagnosis. There was a significant linear trend for patients without diabetes (p-trend=0.002, linear slope of +1.8% per year) as well as patients with diabetes (P-trend=0.002, linear slope of +2.0% per year)


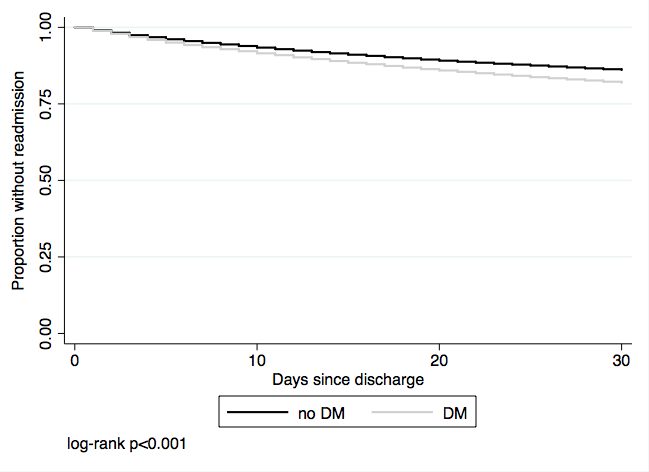


**Supplemental Figure 1:** Cumulative incidence of 30-day all cause readmission among patients hospitalized for a primary diagnosis of atrial firbrilation stratified by diabetes (DM) status.


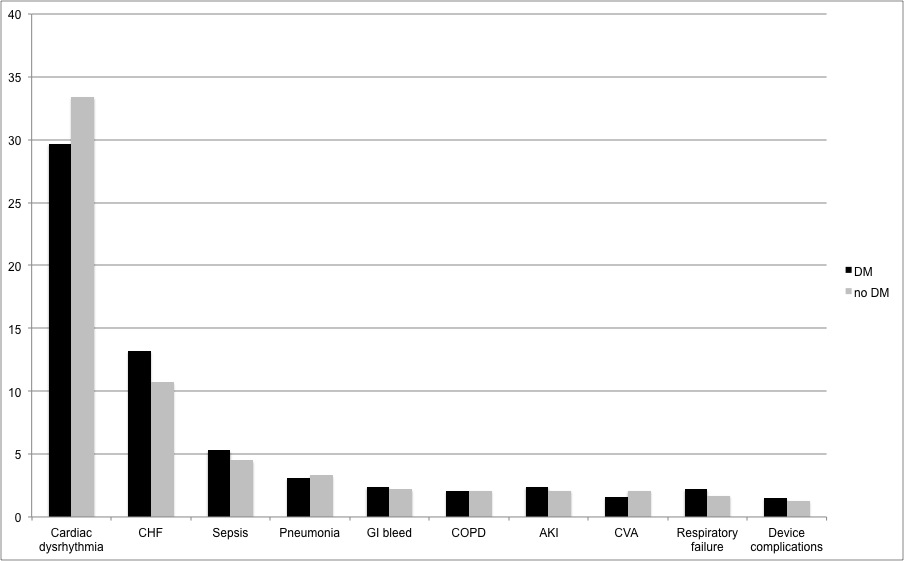


**Supplemental Figure 4:** Ten most common primary diagnoses among patients readmitted within 30-days after discharge of atrial fibrillation hospitalization. When comparing proportions of admissions for specific causes for diabetes vs. no diabetes, P<0.001 for cardiac dysrhythmia, P<0.001 for CHF, P=0.006 for sepsis, p=0.41 for pneumonia, P=0.35 for GI bleed, p=0.98 for COPD, P=0.11 for AKI, P=0.02 for CVA, P=0.008 for respiratory failure, P=0.11 for device complication
